# On the Utilization of Pozzolanic Wastes as an Alternative Resource of Cement

**DOI:** 10.3390/ma7127809

**Published:** 2014-12-05

**Authors:** Md. Rezaul Karim, Md. Maruf Hossain, Mohammad Nabi Newaz Khan, Muhammad Fauzi Mohd Zain, Maslina Jamil, Fook Chuan Lai

**Affiliations:** 1 Department of Civil Engineering, Dhaka University of Engineering and Technology, Gazipur 1700, Bangladesh; 2 Faculty of Engineering and Built Environment, University Kebangsaan Malaysia, Bangi, Selangor 43600, Malaysia; E-Mails: shojib.ce_06@yahoo.com (M.M.H.); nahid.cuet07@gmail.com (M.N.N.K.); mjamil.ukm@gmail.com (M.J.); 3 Sika Kimia Sendirian Berhad, Nilai, Negeri Sembilan Dk 71800, Malaysia; E-Mail: lfcclm@yahoo.com.sg

**Keywords:** slag, palm oil fuel ash, rice husk ash, alkaline activator, alkali activated binder

## Abstract

Recently, as a supplement of cement, the utilization of pozzolanic materials in cement and concrete manufacturing has increased significantly. This study investigates the scope to use pozzolanic wastes (slag, palm oil fuel ash and rice husk ash) as an alkali activated binder (AAB) that can be used as an alternative to cement. To activate these materials, sodium hydroxide solution was used at 1.0, 2.5 and 5.0 molar concentration added into the mortar, separately. The required solution was used to maintain the flow of mortar at 110% ± 5%. The consistency and setting time of the AAB-paste were determined. Mortar was tested for its flow, compressive strength, porosity, water absorption and thermal resistance (heating at 700 °C) and investigated by scanning electron microscopy. The experimental results reveal that AAB-mortar exhibits less flow than that of ordinary Portland cement (OPC). Surprisingly, AAB-mortars (with 2.5 molar solution) achieved a compressive strength of 34.3 MPa at 28 days, while OPC shows that of 43.9 MPa under the same conditions. Although water absorption and porosity of the AAB-mortar are slightly high, it shows excellent thermal resistance compared to OPC. Therefore, based on the test results, it can be concluded that in the presence of a chemical activator, the aforementioned pozzolans can be used as an alternative material for cement.

## 1. Introduction

As a matter of fact, ordinary Portland cement (OPC) is one of the most consumed materials after water. It is used as the main binding material in construction industries across the globe. However, it is an energy intensive material and is also liable for carbon dioxide (CO_2_) gas emission. Thus to reduce consumption and dependency on cement, utilization of pozzolanic materials such as ground granulated blast furnace slag, palm oil fuel ash (POFA), and rice husk ash (RHA), fly ash, silica fume, *etc*. as supplementary cementing materials has become the leading research interest in the area of cement and materials research in recent decades. In addition to CO_2_ emissions, excess fuel is consumed in cement manufacturing for the burning of Portland cement clinker. Besides, it is one of the most energy intensive materials after aluminum and steel [1]. It has been reported that nearly 850–900 kcal/kg (in the dry process) and 1300–1600 kcal/kg (in the wet process) heat energy is required in cement manufacturing [2]. However, the global average electricity consumption is approximately 111 kWh per ton of cement production [3]. Approximately 4.0 kJ energy [4] and 1.5 tons of raw materials are required to produce one ton of OPC. Thus the cement manufacturing process is not only liable for CO_2_ emission but also responsible for the gradual depletion of fuel energy and natural stone from our limited stocks in the planet. Therefore, the acquirement and development of sustainable binding materials is one of the prime issues needed to redress the depletion of the world’s most valuable fossil energy and to reduce the negative impacts of cement production on the environment [5]. On the other hand, huge quantities of RHA, POFA, slag, Fly ash (FA), silica fume, *etc*. are generated regularly worldwide. Table 1 represents a statistics of global production and consumption of various wastes. Table 1 shows that the waste generation rate is high compared to the negligible consumption rate. Only a small portion of slag is consumed as a supplement of cement and an ingredient of concrete [6] and sub-base materials in road construction [7]. Rice husk is consumed for heat generation purposes in rice mills. No promising alternative uses have been developed so far particularly for POFA and RHA and huge costs are incurred for transportation and disposal. Furthermore, enormous environmental pollution is also worth noting.

**Table 1:** A statistic of global production and consumption of various wastes.

Waste	Production Source	Quantity (million ton)	Consumption (million ton)	Reference
Slag	Steel industries	100.00	35	[1]
FA	Coal operated power plants	900.00	-	[8]
POFA	Palm oil mills in Malaysia	0.06	-	[9]
Rice husk	Rice mills	110.00 (20% of 550 million tons rice)	-	[10]
RHA	Rice mills	16.50–22.00	-	[10]
Silica fume	Silicon industries	2.00	-	[8]

Nonetheless, it should be noted that thousands of tons of POFA are produced annually in the operation of 200 palm oil mills in Malaysia and are simply disposed of without any salvage value. So the nation’s pollution problem has increased from this sector which includes the annual production of 2.6 million tons of solid waste in the form of oil palm shells [11]. The production of POFA is growing every year, and is disposed of for landfills, and which now also become a noticeable problem. Besides, similar difficulties have also been faced and noticed in association with the generation of slag and RHA. About 650 million tons of rice is produced in the world of which 2.2 million is from Malaysia; after milling, about 20% is converted to rice husk. Finally, 15%–20% ash is produced as RHA after burning [12]. In spite of their technical and ecological benefits, all of these wastes are simply disposed of into ponds, lagoons or rivers. Therefore, reduction of the quantity of waste dumping, environmental sustainability and declining CO_2_ emission can be ensured by proper consumption or recycling of these materials simultaneously as a supplement of cement or as an ingredient of concrete.

## 2. Research Significance

An investigation into the development of an alternative binder from pozzolanic waste materials (slag, RHA, POFA) is significant as can be seen by the following aspects. Recently, the utilization of pozzolanic materials in cement and concrete has increased considerably due to their diverse benefits such as less cement use, saving production costs, improvement of the durability properties of the concrete and so on. Pozzolans are fine materials that contain silica and/or alumina. They do not exhibit any cementation properties of their own except in the presence of calcium oxide (CaO) or calcium hydroxide (Ca(OH)_2_). Silica and alumina in pozzolans also react and form cementitious materials [13]. POFA is one of the pozzolanic materials that contains a moderate percentage of silicon dioxide and has high potential to be used as a cement replacement material. For example, POFA contains silicon dioxide that can react with Ca(OH)_2_ generated from the hydration process and thus pozzolanic reactions produce more calcium silicate hydrate (C–S–H) gel compound as well as reducing the amount of Ca(OH)_2_. Thus for concrete production, POFA contributes to making concrete stronger, denser and more durable. POFA can also be used to increase both the compressive strength and the sulfate resistance of mortar, if it is ground properly [14]. POFA contributes to improving the durability and is cost effective due to less use of cement. The ground POFA with high fineness can be used as a cement replacement to produce high-strength concrete with a compressive strength as high as 70 MPa at 90 days when used to replace OPC at 20% by weight of binder [15]. Therefore, it will also be beneficial for the environment with respect to reducing the waste disposal volume of landfills. In addition, POFA, RHA and fly ash can be used as pozzolans to replace part of OPC in making mortar with relatively high strength and good resistance to chloride penetration [16]. RHA has been used in lime pozzolan mixes and could be a suitable partial replacement for Portland cement [17,18,19]. RHA is receiving more attention now since its use generally improves the properties of the blended cement concrete, the cost, and leads to a reduction of negative environmental effects [20]. RHA can be incorporated either as an admixture or as cement replacement material [21]. Slag is commonly used in concrete for the following beneficial reasons: it improves durability and reduces porosity, improves the interfacial zone with the aggregate, requires less quantity of cement, saves energy, and exhibits good performance as well as having better engineering properties [22]. Quaternary-blended cement has been produced from slag, POFA, RHA, and timber ash by 66% OPC replacement to be used in high strength (100–120 MPa), sustainable, and high-performance concrete [23].

Therefore, effective consumption of these waste materials as a replacement for cement will also encourage researchers to investigate a sustainable way of saving material, especially cement as well as reducing the CO_2_ emission. In addition, the utilization of these waste materials will have the following multiple advantages: enhances the properties of concrete, reduces the production cost of concrete, and minimizes the waste disposal problem. Finally, use of these wastes as a supplement of cement and or as an ingredient of concrete is logical, worthy and attributable to the present situation which demands the achievement of a goal of a sustainable concrete and sustainable binding material. Thus, expectation for the development of an alternative binder from local waste materials is realistic and meaningful. The aim of this research is, therefore, to investigate the possibility of producing an alkali activated binder (AAB) using 100% local industrial slag, RHA and POFA by a mechano-chemical (grinding and chemical activators) activation technique.

## 3. Materials and Methods

### 3.1. Materials

Ground granulated blast furnace slag, POFA and RHA were used in this research as components of AAB. OPC (American Society for Testing and Materials (ASTM) type I) was used to compare the different properties including physical, chemical, binding, flow, compressive strength, microstructure and durability of the AAB. Slag and POFA were collected from local industries, Selangor, Malaysia. RHA was produced by a special type of furnace at the concrete lab at the University of Kebangsaan Malaysia (Bangi, Selangor, Malaysia). The details of the furnace were reported by Zain *et al.* [24]. Local river sand which passed through a 4.75 mm sieve and had a specific gravity of 2.62 was used as fine aggregate. Sodium hydroxide (NaOH) flakes of analytical grade were used as a chemical activator from the manufacturer Merck (Hunterdon, NJ, USA). DarexSuper-20 was used as superplasticizer (SP) from the Grace manufacturing company (Russellville, AR, USA). Though the use of higher amounts of SP has a negative effect on the overall cost and eco efficiency of the materials it was used to increase and maintain a sufficient flow for casting of mortar. SP is considered as grade F according to ASTM C494 specification [25]. Available supplied water was used for mortar preparation and in curing purposes.

### 3.2. Instruments

The following instruments were utilized to perform different experiments in the research: Malvern Mastersizer 2000 (for grain size analysis, Malvern, UK); X-ray fluorescence (XRF) was done for chemical composition using a Bruker brand (Billerica, MA, USA) XRF machine. A Los Angeles abrasion machine (ELE International Limited, London, UK) was used for grinding of POFA and RHA for increased fineness. X-ray diffraction (XRD) analysis was conducted using a Bruker brand XRD machine. Scanning electron microscope (SEM) analysis was performed by Supra 55 VP, ZEISS (Oberkochen, Germany). A Hobart (Troy, OH, USA) mixing machine was used to mix the paste and mortars. A compression machine (Unit Test scientific, Sendirian Berhad, Selangor, Malaysia) of 3000 kN capacity was used for compressive tests of mortar specimens. An electric furnace was used for thermal testing.

### 3.3. Preparation of Mortar

Table 2 presents the mixing proportions of the raw materials (slag, RHA and POFA). Paste and mortar were prepared according to ASTM C305-06 specification using a Hobart mixing machine [26]. NaOH solutions of different molar concentrations (1.0, 2.5 and 5.0 M) were prepared before mixing/preparation of mortar. Compaction of the mold was performed using an electrically operated vibration table. After casting, the prism mold of OPC was opened after one day but molds of AAB-mortars for 1.0 M concentration needed two days because of delayed hardening. Finally, samples were immersed into a curing tank at a room temperature of 25 ± 20 °C until the desired testing ages of 3, 7 and 28 days were achieved.

**Table 2:** Materials used for preparation of mortar (by weight).

Binder			Activator	Molar concentration	Solution to binder or water to cement ratio	SP (%)	Sand to binder ratio
Name	Materials	(%)					
AAB	Slag	70	NaOH	1.0 M	0.62	4.2	2.75
	POFA	20		2.5 M		4.6	
	RHA	10		5.0 M		5.0	
OPC	-	100	-	-	0.55	2.5	

### 3.4. Tests on Paste and Mortar

#### 3.4.1. Normal Consistency of Binder

Consistency (water demand) of AAB and OPC paste was determined according to ASTM C187-04 [27]. In this regard, pastes were prepared and the amount of water required noted to penetrate the Vicat testing (ELE International Limited, London, UK) needle (10 mm diameter) by 10 mm.

#### 3.4.2. Setting Time of Binder

For the observation of the setting time, the ASTM C191-08 testing standard was followed [28]. The Vicat apparatus was also used to determine the setting time of the AAB as well as the OPC paste. The initial setting time was measured for standard paste (prepared using the required water according to the normal consistency) to penetrate the Vicat testing needle (1 mm diameter) by 25 mm. The final setting time was the measured time when there was no penetration of the needle observed by the same testing method.

#### 3.4.3. Flow and Compressive Strength of Mortar

The mortar flow spread test was done using a flow table according to the ASTM C1437 testing standard [29]. The compressive strength and the strength activity index of mortar were determined using a 50 mm cube specimen according to the ASTM C109 testing standard [30].

#### 3.4.4. Water Absorption of Mortar

Water absorption was determined using a mortar prism of 40 mm × 40 mm × 160 mm in size with specimens following the Japanese industrial standard (JIS A6203) as found in a study by Ahmad *et al*. [31]. In this regard, prism specimens aged for 28 days were dried until constant weight (*W*_d_) was achieved. Then the specimens were immersed into water. Subsequently, the specimens were taken out and their surfaces wiped quickly with a wet cloth. Then they were weighed in air immediately (*W*_a_) after immersion periods of 30 min, 1, 3, 6, 24, 48 and 72 h. Thus, water absorption of the specimen was calculated as 100 × (*W*_a_ – *W*_d_)/*W*_d_.

#### 3.4.5. Porosity of Mortar

Porosity of mortar was determined with a prism of 40 mm × 40 mm × 160 mm in size of specimens based on the study of Chindaprasirt *et al.* [32]. After being cured in water until the age of 28 days, prism specimens were taken. The specimens were dried at 100 ± 5 °C until they achieved a constant weight (*W*_d_). Then, the specimens were immersed in clean water for full saturation over three days. After that, the weights of the specimens in water were also recorded (*W*_w_). Subsequently, the specimens were taken out and their surfaces wiped quickly with a wet cloth. Specimens were weighed in air immediately (*W*_a_). Therefore, the porosity of the specimens was obtained in percent as 100 × (*W*_a_ − *W*_d_)/(*W*_a_ − *W*_w_).

#### 3.4.6. Thermal Resistance of Mortar

For the heat resistance test, using a prism of 40 mm × 40 mm × 160 mm in size, specimens were weighed at the saturated surface in dry condition (*W*_s_). Then, the specimens were put into an electric furnace and heated up to 700 °C for two hours with the temperature increment of the furnace at 9 °C per minute. After completion of the heating period, specimens were cooled to room temperature over approximately the same period of time. Subsequently, the dry weight of the specimens was taken as *W*_D_ and the strength of the specimens was determined (*f*_cf_). Finally, loss in weight was calculated as (*W*_s_ – *W*_D_)/*W*_s_ and the strength loss of the specimens due to the thermal effect was determined as (*f*_c_ − *f*_cf_)/*f*_c_; where, *f*_c_ is the strength of the ordinary sample (without being heated). The same study was successfully performed by Rahel *et al.* [33].

## 4. Results and Discussion

### 4.1. Properties of Materials

#### 4.1.1. Chemical and Physical Properties of Materials

The chemical and physical properties of materials are represented in Table 3. To determine the chemical properties, the raw materials and the new binders were examined by X-ray fluorescence (XRF) test. The Table 3 shows that among all types of raw materials, RHA contains the greater amount of silica (87.75%), which is responsible for the pozzolanic reaction or secondary hydration in mortar or concrete. The total percentage of major oxides (SiO_2_ + Al_2_O_3_ + Fe_2_O_3_) is over 70% for RHA which is greater than the minimum (70%) as specified in ASTMC618 [34]. Thus, RHA is categorized as class F pozzolan. While, the sum of these oxides lies between 50% and 70% both for slag and POFA, accordingly, they may be considered as class C pozzolan. Since POFA does not possess any cementing property, it cannot be categorized as class C pozzolan. The sulphur trioxide (SO_3_) content of these materials is less than 4% which is the maximum allowable content as prescribed by the ASTM C618 standard [34]. POFA contains 11.86% of K_2_O which is greater when compared to the other materials, because palm oil trees consume a large amount of potassium from the soil during cultivation.

The loss on ignition (LOI) is higher for POFA, but it is less than the prescribed value 10% (ASTM C-618) [34]. The physical properties of the materials are also given in Table 3. The particle size of POFA and RHA decreases after grinding. The average particle sizes of the materials were found to be 14.67 µm for slag, 16.08 µm for POFA, 6.63 µm for RHA and 16.17 µm for OPC. Thus, the raw materials have a lower particle size in comparison to OPC. Specific gravity and fineness of the particles are also shown in Table 3 which shows that pozzolanic materials are lighter than OPC. Similar observations were reported by past researchers [35].

**Table 3:** Chemical and physical properties of materials.

**Material**	**Chemical Properties, Oxide Compositions (%)**											
	SiO_2_	Al_2_O_3_	Fe_2_O_3_	CaO	MgO	SO_3_	Na_2_O	K_2_O	P_2_O_5_	TiO_2_	MnO	LOI
Slag	33.05	16.36	0.53	45.00	6.41	1.21	0.13	0.42	-	-	-	3.05
POFA	47.22	2.24	2.65	6.48	5.86	3.34	1.22	11.86	5.37	0.17	0.10	5.42
RHA	87.75	0.38	0.19	1.04	0.69	0.56	0.05	2.83	1.31	0.02	0.07	3.04
OPC	20.99	4.60	4.44	67.17	2.53	2.98	0.03	0.16	-	-	-	1.30
AAB *	41.35	11.94	0.92	32.90	5.73	1.57	0.34	2.95	1.21	0.04	0.03	3.52
	**Physical Properties**											
	Specific gravity	Average grain size d_50_ (μm)			Fineness						Color	
					Blaine (cm^2^/g)		Retained on 45 μm sieve (%)					
Slag	2.85	14.67			3,919		0.14				Near white	
POFA	2.16	16.08			4,582		4.23				Blackish white	
RHA	2.05	6.63			6,964		3.32				Near white	
OPC	3.14	16.17			2,850		12.52				Grey	
AAB *	2.63	14.15			4,356		1.28				Near white	

* Calculated Value; AAB = 70% Slag + 20% POFA + 10% RHA.

#### 4.1.2. SEM Images of Materials

**Figure 1:**
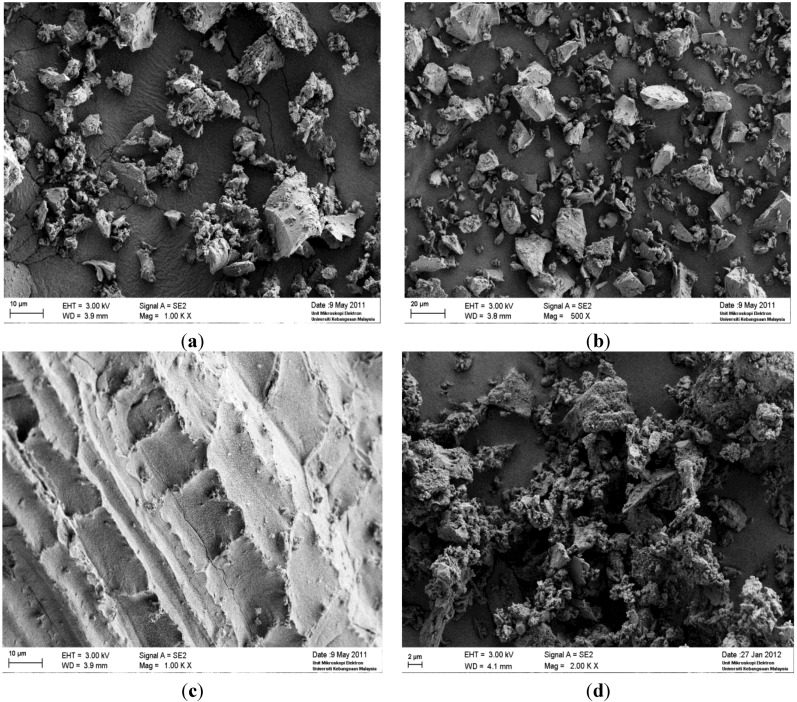

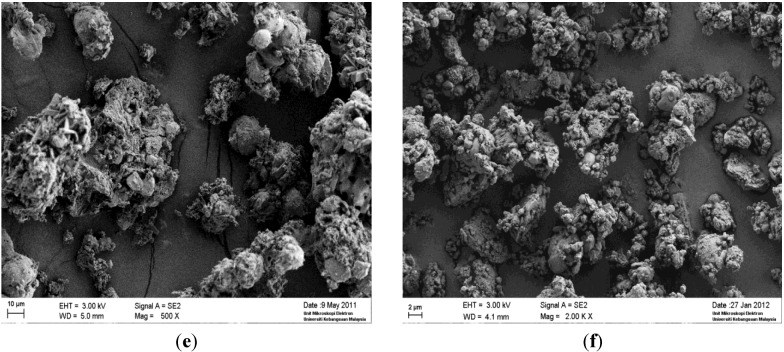
SEM image of (**a**) OPC; (**b**) Slag. (**c**) RHA (as produced); (**d**) RHA (after grinding); (**e**) POFA (as received); and (**f**) POFA (after grinding).

The obtained SEM views of the used raw materials are shown in Figure 1. Slag particles, when seen with a SEM image, appear to be square and diamond in shape and similar to the particles of OPC. The OPC seems to have box and stone-shaped particles observed under SEM. The original RHA shows a spongy and cellular structure. The original POFA (as received) shows that it contains close to spherical particles with a small amount of plerospheres and an irregularly shaped porous cellular structure. After grinding POFA and RHA, the porous structures were crushed and broken down into smaller fractions leading toan increased surface area and improved fineness of the particles.

#### 4.1.3. Strength Activity Index of Binders

ASTM C 311 defines the strength activity index (SAI) as the ratio between the compressive strength of mortar containing substitute materials of 20% by mass of binder and that of the average compressive strength of the reference cement (OPC) mortar at a designated age [36]; but the material replacement is 50% for the SAI of slag. The SAI of the used materials was determined and the obtained values are presented in Table 4. Experimental results show that the activity index of slag is more than 100% for both 7 and 28 days. Thus, slag is considered as 100 grade based on the ASTM C 989-05 classification [37]. Activity indexes of unground RHA and POFA are below 50% and over 54% at 7 days respectively. They are found to be more than 63% at 28 days. Activity indexes of RHA and POFA are found to be less, due to the use of unground POFA and RHA in preparing testing mortar samples. Usually, the strength activity index of any pozzolans can be improved by grinding or increasing their fineness as can easily be seen from Table 4 as well as found in past studies [38,39]. Thus, the activity index of GPOFA (ground POFA) and GRHA (ground RHA) is much higher than that of the unground sample. Therefore, pozzolans can be further activated by improving their fineness.

**Table 4:** Strength activity index (SAI) of materials.

Binder	SAI (7 days)	ASTM requirement	SAI (28 days)	ASTM requirement
Slag	100.4	95 for 120 grade	103.8	95 for 100 grade
RHA	48.6	-	63.0	-
GRHA	86.7	-	101.6	-
POFA	54.2	-	65.3	-
GPOFA	84.9	-	99.0	-

### 4.2. Consistency of Binders

Table 5 gives the consistency values of the binders. The Table 5 indicates that the consistency of OPC is 30.0%. In contrast, AAB pastes show high consistency (water demand) compared to OPC (consistency for AAB pastes is 33.5%). This increased consistency occurs due to porous and spongy particles, the fineness and more specific surface areas of the pozzolans, as observed in SEM analysis. Ganesan *et al*. [40] reported that the consistency of RHA blended pastes gradually increases due to the addition of RHA to the paste. Therefore, it can be understood that a blended paste of pozzolanic materials show a greater consistency than that of OPC pastes. This argument is also supported by Cheerarot *et al*. [41] who reported that the normal consistency of OPC paste is lower than that of the blended paste of FA.

**Table 5:** Consistency, setting time and flow of binder.

Binder	AAB (1.0 M)	AAB (2.5 M)	AAB (5.0 M)	OPC
Consistency (%)	33.5	33.5	33.5	30.0
Initial setting time (hour:min)	0:50	0:27	0:21	2:15
Final setting time (hour:min)	2:10	1:45	1:05	5:25
Flow (%)	114	110	106	109

### 4.3. Setting Time

The setting time of the binders is presented in Table 5. The setting time of the AAB paste is earlier compared to OPC and it completely depends on the concentration of the NaOH solution, the percentage of raw materials/ingredient of AAB mix and the fineness (unground and ground conditions) of POFA and RHA. For instance, on increasing the molar concentration of NaOH solution, the setting time of the binders gradually decreased because AAB contains 1.0, 2.5 and 5.0 M solutions respectively. The initial setting time of OPC pastes was found to be 2 h and 15 min. The final setting time of AAB paste was observed to be less than that of the value for OPC, as specified in ASTM C150 [42]. However, Tangchirapat *et al*. [43] reported that the setting time of the paste increases after OPC replacement by POFA. For the present study, the setting time of AAB (100% replacement of OPC) is less which may be due to the chemical reaction of NaOH and pozzolans. NaOH contributes to the earlier setting of the AAB because of its smaller cation (Na^+^). This is according to Fajan’s rule: (i) the more charged the cation is, the closer and stronger it will pull other molecules to it; (ii) The smaller the cation, the less the levels of electron shielding get in the way, letting other molecules be pulled closer and stronger [44].

### 4.4. Flow of Binders

The flow of the binders is presented in Table 5. The flow of the OPC mortar is 109% only when using 2.5% super plasticizer. However to obtain the same value of flow, over 4% SP and a greater amount of water solution was used in the case of the AAB-mortar. NaOH in pozzolans contributes to gaining strength quickly and a resultant lesser flow which is a similar argument to that reported by Jae *et al*. [45] .The lesser flow of mortar containing NaOH may be due to the following reasons:

1. NaOH has the smallest cation (Na^+^), which may attract the molecules/constituents of the binder and the mortar quickly and consequently, reduce the flow of mortar.
2. The lower flow tendency and the higher water demand are due to the porous and spongy nature of pozzolanic materials (particularly for RHA) and the higher fineness or larger surface area; thus a greater amount of water is required. Several researchers reported that a greater amount of water was required to obtain the desired consistency and a lower flow ability is common among pozzolans as reported by Ahmad and Sheikh [46].

### 4.5. Compressive Strength of Mortar

**Figure 2:**
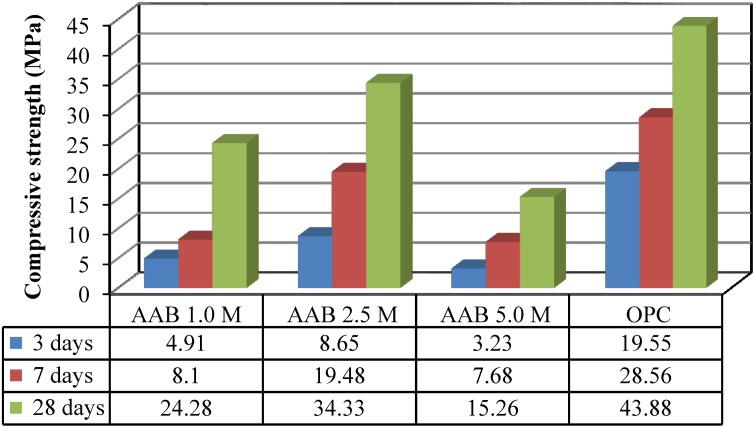
Compressive strength of mortar.

In the current study, a compressive strength of 34.3 MPa was found using slag, POFA and RHA with only 2.5 M NaOH solution and an ambient temperature curing method. This better strength development of mortar was also observed by Isaia *et al*. [47] in binary and/or ternary combination due to the synergic effect of the materials. The compressive strength of AAB-mortars for a different activator concentration is presented in Figure 2. The figure shows that the strength of mortar is influenced significantly by the concentration of activators which is a similar conclusion as reported by other researchers [48,49]. The compressive strength of AAB-mortar has a less value with 1.0 molar (1.0 M) solution at all ages. On the contrary, the strength of all AAB-mortar is less than that of OPC. However, the strength of AAB-mortar is better with 2.5 M solution but worsens for the 5.0 M solution. This reduced strength of mortar obtained may be due to the following reasons:

1. A lower concentration (1.0 M) may not be sufficient to activate all of the molecules of AAB.
2. For a higher molarity (5.0 M), excess amounts of activators remain without being bonded or form weak intra-bonds inside the AAB-mortar.
3. Residual activators or weak bonds of activators may collapse when the mortar is immersed in water for curing. As a result, the strength of the mortar remains the same or decreases.

The AAB-mortar achieves the highest strength with 2.5 M activators. Therefore, based on experimental results, NaOH contributes towards developing the highest strength of the AAB-mortar with 2.5 M solutions at all ages. It could be noted that the compressive strength of AAB-mortars containing 2.5 M solution is better and so the morphology and durability properties of these mortars were investigated.

### 4.6. SEM Observation of Mortar

**Figure 3:**
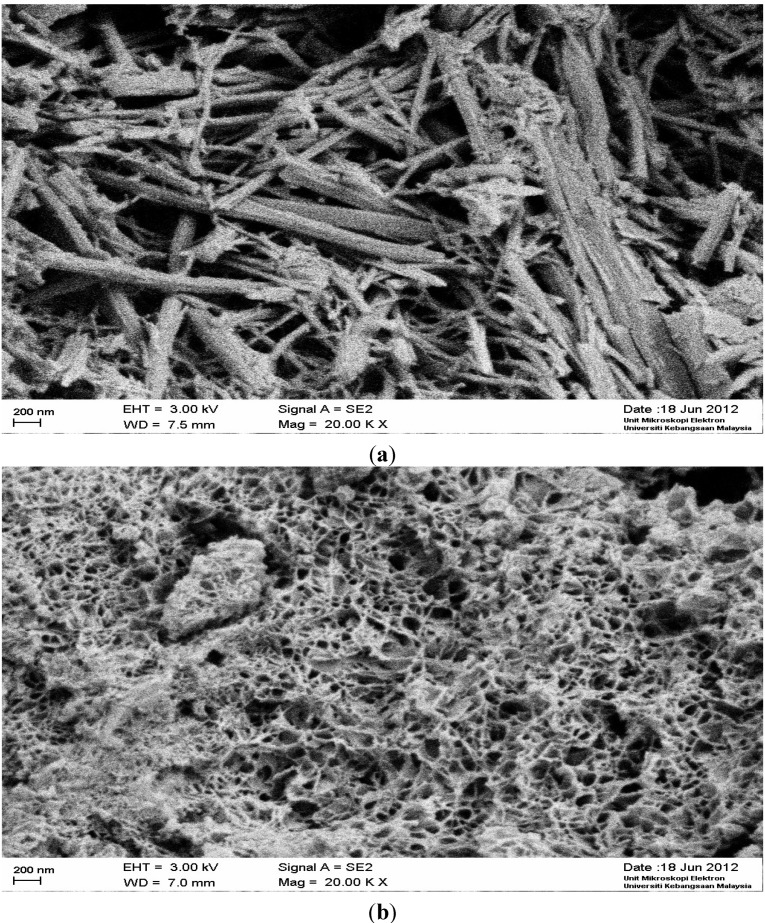
SEM views of (**a**) OPC; (**b**) AAB-mortar.

SEM images of AAB exhibit a honeycomb type heterogeneous gel structure with embedded varying morphologies associated with hollow cavities, likened to a geopolymer structure. A similar argument was reported by Rodriguez *et al*. [50] for an alkali activated binder. From the SEM images (Figure 3), large differences can be observed from the microstructures of OPC and AAB-mortars. In comparison to the shape of the gel formation, the image of OPC shows the existence of ettringite (needle type shape) while that of AAB demonstrates a different view. There is no ettringite observed in the SEM analysis of AAB-mortars, it contains amorphous and a sponge-type gel structure. Therefore, the strength formation of AAB-mortar and that of OPC seems to be completely different.

### 4.7. Water Absorption of Mortars

**Figure 4:**
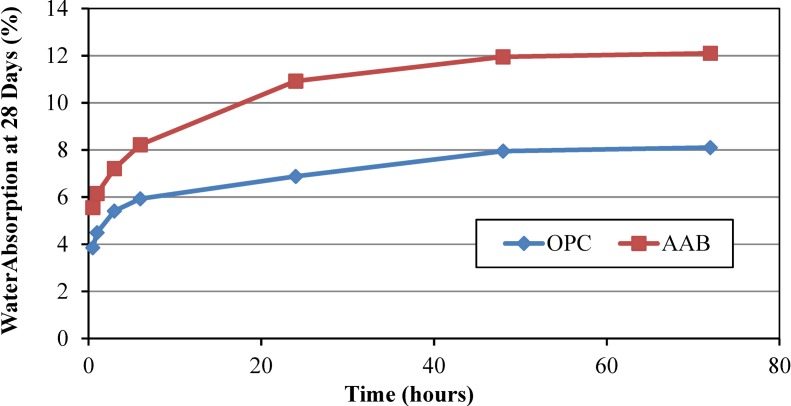
Water absorption of AAB and OPC mortar at 28 days of curing age.

Figure 4 represents water absorption of OPC and AAB mortar at 28 days of curing age. It was found that water absorption of AAB mortar was over 12%; while, that obtained for OPC mortar was 8.1%, tested after an immersion period of 72 h at 28 days. It is interesting to note that the rate of water absorption is lower at the beginning of the immersion period (*i.e.*, within 30 min to 3 h) at 28 days of testing age. Water absorption of AAB-mortar is higher than that of OPC mortar. The absorption of AAB-mortar was found to be higher due to more voids and pores (Figure 3) present inside the mortar which also lead to a lower compressive strength of the mortar. In addition, AAB has finer particles than those of OPC; consequently, they absorb more water which can be observed from normal consistency data (Table 5).

In general, for the case of partial replacement of slag, POFA, and RHA results in pore refinement and increased water tightness of the binder matrix. The refinement of the pore structure, however, leads to reduced permeability of the hydrated cement paste and this could lead to a retarded moisture migration through the cement matrix [51]. For the alkali activated binder, Chi and Huang [52] obtained water absorption of 7.5% for an OPC specimen; however, absorption was reduced significantly for the alkali activated binder (1.1%–6.1%); it depends on the ratio of slag-to-fly ash and activator content. This high performance of the water absorption was obtained most probably due to the high compressive strength (20–80 MPa) of mortar.

### 4.8. Porosity of Mortars

The average porosity of mortar specimens was determined at the age of 28 days. The porosity of OPC and AAB mortar was found as 14.23% and 20.54% respectively. The higher porosity of AAB mortars was found to be due to the small particle size, the siliceous admixture (POFA, RHA and slag) particles have more surface area hence absorb greater amounts of water and have lower compressive strength. A similar observation was reported by Chindaprasirt and Rukzon [32]. In addition, more voids are occupied inside the AAB mortar; the situation can be seen from the SEM image.

However, in the case of partial replacement, incorporation of RHA reduces the porosity [19,51]. Inclusion of FA reduces the average pore size and results in a less permeable paste as reported by Chindaprasirt *et al*. [53]. It was also found by Chindaprasirt *et al*. [54] that the permeability of rice husk-bark ash and POFA are lower than that of OPC concrete. Al-Otaib [55] reported that alkali activated slag concrete shows a greater porosity value compared to OPC. He obtained a range of porosity of 13%–10% for 7–360 days of testing time depending on the slag replacement, while OPC concrete showed a porosity of 10.4%–8% for the same age. The main variable to be considered, for obtaining long lasting concrete, is porosity and pore connectivity [56]. Higher porosity and absorption can be reduced by achieving a higher compressive strength of the AAB mortar [32].

### 4.9. Thermal Resistance of Mortar

**Figure 5:**
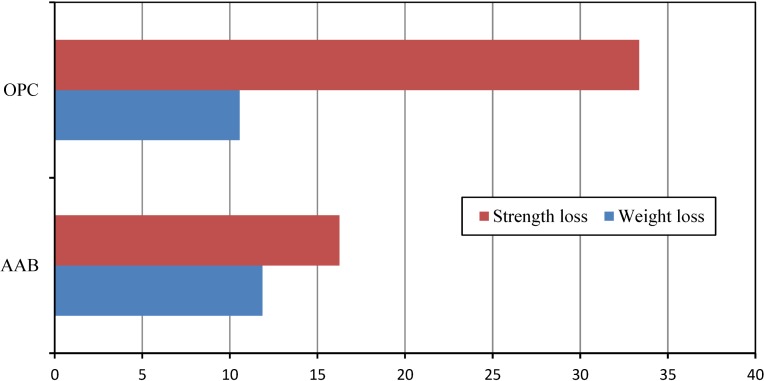
Losses (%) in weight and strength of mortar after heating at 700 °C.

After heating the mortars at 700 °C, the weight loss of OPC samples was found to be 10.56%, while the weight loss of the AAB-specimen obtained was a little over 11.86%. Loss in strength of OPC and AAB-mortar was found as 33.35% and 16.26%, respectively (Figure 5). Although Rahel *et al*. [33] reported that the strength loss of OPC mortar is 32% after being heated at 700 °C. Alkali activated binders (slag or slag with metakaolin blended) are very good against thermal resistance up to 800–1400 °C as reported by several studies [57,58,59]. In our present study, AAB mortar exhibits a very strong thermal resistance compared to that of OPC up to exposure at 700 °C. This may have occurred due to the strong bond of the NaOH in AAB mortar and the higher boiling point (1390 °C) of NaOH. Thus, the strength of AAB mortar did not reduce significantly due to exposure at 700 °C.

## 5. Conclusions

Based on the experimental test results, the following conclusions can be drawn from the present study:

1 The physical and chemical test results reveal that the considered pozzolanic waste materials (slag, POFA and RHA) contain a high amount of silica and sufficient amounts of the major oxides. Consequently, they perform as an alternative binder in the presence of a chemical activator, NaOH. It can be predicted that development of a new binder from locally available slag, POFA and RHA might well be possible.
2 Experimental results revealed that slag, POFA and RHA could be used as substitutes of cement provided that these wastes are processed properly with maintenance of high fineness and with the use of a chemical activator.
3 The alternative binder exhibits reasonable binding, consistency, flow value and setting time compared to OPC. The new binder shows a considerable compressive strength of 34.3 MPa at 28 days.
4 The water absorption and porosity of the AAB mortar are slightly higher compared to those of OPC mortar. This occurs due to the porous structure and the less compressive strength of AAB. It can be minimized by improving the strength of AAB.
5 An excellent thermal resistance was found with the AAB-mortar when it was exposed to 700 °C for two hours. Only 16.26% of the strength was reduced in the case of AAB-mortar while OPC lost more than 32% of its strength under the same conditions.
6 Furthermore, as an alternative material of cement, the consumption of slag, POFA and RHA in the presence of NaOH would be a probable and sustainable solution to reduce the demand of cement which also helps to achieve the goal of sustainable concrete.
